# IOS-1002, a Stabilized HLA-B57 Open Format, Exerts Potent Anti-Tumor Activity

**DOI:** 10.3390/cancers16162902

**Published:** 2024-08-21

**Authors:** Anahita Rafiei, Marco Gualandi, Chia-Lung Yang, Richard Woods, Anil Kumar, Kathrin Brunner, John Sigrist, Hilmar Ebersbach, Steve Coats, Christoph Renner, Osiris Marroquin Belaunzaran

**Affiliations:** 1 ImmunOs Therapeutics AG, 8952 Schlieren, Switzerland; 2 ACROBiosystems, 4056 Basel, Switzerland; 3 Department of Biomedicine, University Basel, 4031 Basel, Switzerland

**Keywords:** HLA open formats, human leukocyte immunoglobulin-like receptor, killer immunoglobulin-like receptor, innate checkpoint blockade

## Abstract

**Simple Summary:**

The human leukocyte antigen (HLA) system is crucial for immune responses against infections and cancer. Certain HLA class Ia molecules, like HLA-B27 and HLA-B57, are linked to both viral control (e.g., HIV, HCV) and development of autoimmune diseases (e.g., Spondylarthritis and psoriasis, respectively). Here we explore the use of HLA class I molecules as therapeutics in an “open format” (peptide free) construct, designed to activate immune cells through the interaction of LILRB and KIR receptors. The study identified IOS-1002, a modified version of HLA-B57:01:01 molecule fused to an IgG4 engineered to enhance stability and manufacturability. The research described in this manuscript highlights IOS-1002’s unique mechanism of action, which includes binding to LILRB1, LILRB2, KIR3DL1 and CD64 receptors leads to the stimulation of multiple effector cells from both the innate and adaptive immune system. IOS-1002 is a first-in-class, multi-target, and multi-functional agent, currently being evaluated in a first-in-human phase I trial for various solid tumor indications.

**Abstract:**

HLA-B27 and HLA-B57 are associated with autoimmunity and long-term viral control and protection against HIV and HCV infection; however, their role in cancer immunity remains unknown. HLA class I molecules interact with innate checkpoint receptors of the LILRA, LILRB and KIR families present in diverse sets of immune cells. Here, we demonstrate that an open format (peptide free conformation) and expression- and stability-optimized HLA-B57-B2m-IgG4_Fc fusion protein (IOS-1002) binds to human leukocyte immunoglobulin-like receptor B1 and B2 (LILRB1 and LILRB2) and to killer immunoglobulin-like receptor 3DL1 (KIR3DL1). In addition, we show that the IgG4 Fc backbone is required for engagement to Fcγ receptors and potent activation of macrophage phagocytosis. IOS-1002 blocks the immunosuppressive ITIM and SHP1/2 phosphatase signaling cascade, reduces the expression of immunosuppressive M2-like polarization markers of macrophages and differentiation of monocytes to myeloid-derived suppressor cells, enhances tumor cell phagocytosis in vitro and potentiates activation of T and NK cells. Lastly, IOS-1002 demonstrates efficacy in an ex vivo patient-derived tumor sample tumoroid model. IOS-1002 is a first-in-class multi-target and multi-functional human-derived HLA molecule that activates anti-tumor immunity and is currently under clinical evaluation.

## 1. Introduction

Background and hypothesis: The human leukocyte antigen (HLA) system determines the capability of the immune system to develop an adaptive cellular immune response against infections and cancer. For the classical HLA class Ia (HLA-A,-B,-C) molecules, a trimer of HLA molecule, beta-2-microglobulin (B2m) and peptide is formed and presented to peptide-specific CD8^+^ T cells [1]. However, there are examples of certain HLA class Ia molecules, such as HLA-B27 or HLA-B57, where superior control of viral infections (e.g., HIV and HCV) and autoimmune predisposition is mediated by immune cell activation [2,3,4], specifically by CD8^+^ T cells, but is independent of epitope and peptide specificity [4,5]. Neither the mechanisms of viral control nor the pathways involved in loss of control leading to disease progression are well characterized in such settings. It has been hypothesized that the possession of these alleles and expression of HLA heavy chains lacking peptide, with or without B2m (described here as HLA open format), may contribute to viral control and enhanced risk of autoimmune-associated diseases (e.g., Spondylarthritis (SpA) for HLA-B27 and psoriasis for HLA-B57) [3,5,6]. The role of HLA-B27 in SpA development has been extensively studied and the prevailing hypothesis relies on the observation that HLA-B27 open formats are expressed on the immune cell surface of patients possessing the HLA-B27 allele [3,7]. Multimerization of HLA-B27 open formats has been associated with immune cell activation and development of autoimmune disease through increased receptor interaction [7]. In viral control, individuals carrying protective alleles (e.g., HLA-B27, HLA-B57) while maintaining low viral loads are so called long-term non-progressors (LNTPs). The prevailing hypothesis in LNTPs is that the presentation of viral peptides by MHC to CD8^+^ T cells lead to specific immune cell responses and control of infected cells [2]; however, alternative mechanisms independent of epitope and peptide may lead to immune cell activation and viral control through the interaction of HLA to LILRB and KIR receptors [2,4,5,8,9].

Receptor interactions and mechanisms: Key target receptors for HLAs have been identified within the human leukocyte immunoglobulin-like receptor (LILR) and human killer cell immunoglobulin-like receptor (KIR) family, respectively [10]. Human leukocyte immunoglobulin-like receptor subfamily B1 and 2 (LILRB1/LILRB2) are inhibitory checkpoint receptors that have long cytoplasmic tails of immunoreceptor tyrosine-based inhibitory motifs (ITIM) that, upon activation, lead to the recruitment of SHP protein tyrosine phosphatases that impact cell growth, migration, invasion, differentiation, survival and cellular trafficking [11]. A diverse array of ligands has been reported to interact with LILRB1 and LILRB2 receptors. The LILRB1 receptor ligands include classical and non-classical MHC class I molecules such as HLA-G1, UL18 (a cytomegalovirus/Major Histocompatibility Complex (MHC)-I homolog), calcium-binding proteins, S100A8/9 and RIFIN proteins [11]. LILRB1 is expressed on monocytes, macrophages, natural killer (NK) cells, T cells, B cells, dendritic cells (DCs), eosinophils and basophils [12]. Ligands for LILRB2 include classical and non-classical MHC class I molecules such as HLA-G1 and HLA-G2, Angiopoietin-like proteins (ANGPTL 2/7), myelin inhibitors (including Nogo66, MAG and OMgp) [12], β-amyloid and SEMA4A [11]. LILRB2 is expressed on monocytes, macrophages, DCs, neutrophils, basophils, hematopoietic stem cells and platelets [12]. Mouse orthologs of LILRB molecules are paired immunoglobulin-like receptor B (PirB) [13] and gp49B1 [11] expressed by cells of the myeloid compartment (monocytes, macrophages, DCs and granulocytes) [11]. Studies in PirB knock-out mice demonstrated an enhanced anti-tumor response leading to reduced tumor burden [13] which underlies evidence of blocking human LILRB receptors for anti-tumor activity.

The LILR family shares similarities to the human killer cell immunoglobulin-like receptor (KIR) family, with notable homologies in both their cytoplasmic domains and Ig-like structures [14]. Ligands of KIR3DL1 are HLA-A and HLA-B allotypes that possess the Bw4 epitope. Expression of KIR3DL1 is restricted to NK cells and subsets of T cells. KIR3DL1 receptors are known to have a dual role in that they possess intracellular inhibitory ITIM signaling motifs capable of recruiting SHP1/2 phosphatases and yet are also capable of triggering NK cell cytotoxic responses by detecting diseased cells that have absent or altered expression of HLA class I molecules (e.g., cancer cells) in a process termed licensing or education [15]. In addition, the association of KIR3DL1 and HLA-B57 with human immunodeficiency virus (HIV) protection has been extensively documented [16].

Study objective: Based on the function of HLA-open formats in autoimmune diseases, we performed a systematic analysis of different HLA class I open formats fused to an IgG4 fragment crystallizable (Fc) molecule to increase the interaction by avidity with diverse LILRBs, KIRs and Fcγ receptors to block receptor-mediated signaling with the goal to develop them as cancer therapeutics. Through this process, we identified HLA-B57:01:01.B2m open format as a very promising candidate and demonstrate that an optimization by 2 point mutations resulted in a lead candidate (HLA-B57^(A46E/V97R)^.B2m-IgG4_Fc, called IOS-1002) with superior expression and with specific and avidity-driven binding to LILRB1, LILRB2, KIR3DL1 and CD64 and finally to a potent modulation and activation of immune cells. IOS-1002’s ability to bind with low nanomolar affinity to CD64 provides IOS-1002 with a unique ability to induce macrophage phagocytosis. In summary, IOS-1002 acts as regulator of innate and adaptive immune surveillance and is currently in phase I clinical trial in patients with advanced stage solid organ cancers.

## 2. Materials and Methods

### 2.1. In Silico Model of HLA-B57 Structure

The model of the HLA-B57 depicting the mutation sites A46 and V97 in HLA-B57^(A46E/V97R)^ was constructed in PyMOL (version 2.3.4) using the template structure of HLA-B57:01:01 obtained from RCSB database (PDB: 5VUF). Structural superimposition of the HLA-B57 structure (PDB: 2HJK) on LILRB1/HLA-G (PDB: 6AEE) and LILRB2/HLA-G (PDB: 2DYP) complex structures was performed using CCP superpose program. The figures were generated using PyMOL software suite (version 2.3.4) [17,18].

### 2.2. Viability and Protein Productivity Assessments

Multiple constructs of HLA-B57 or HLA-B57^(A46E/V97R)^ were stably co-transfected with and without B2m at different ratios into CHO cell lines and the respective cell viability and protein titer productivity of the transfected constructs were quantified. The cell viability and cell densities per unit volume were determined using Vi-Cell Counter (Beckman Coulter, Brea, CA, USA) and the expressed protein titers were quantified using Octet Red96 system (Sartorius, Göttingen, Germany) using protein A biosensors (Sartorius, Göttingen, Germany, 18-5082).

### 2.3. Construction, Expression and Purification of Recombinant Proteins

The recombinant proteins used in the study were constructed as either monomers (His-tagged) or dimers (human IgG4Fc or mouse IgG2aFc tagged). The proteins were either stably or transiently expressed in CHO cell line. Proteins were purified from supernatants using affinity purification followed by size exclusion chromatography (SEC) using GE AKTA Purifier 10 FPLC System with a HiLoad 16/600 Superdex (Cytiva, Marlborough, MA, USA, 28-9893-35) or Superdex 200 10/300 (Cytiva, Marlborough, MA, USA, 28-9909-44). Affinity purification was performed by either using Protein G Sepharose beads (Sigma, St. Louis, MO, USA, GE-17-0618-01) or HiTrap PrismA protein A 0.4 mL chromatography column (Cytiva, Marlborough, MA, USA, 17549856) or using Ni-NTA based affinity purification. The purified proteins were subsequently concentrated using Amicon Ultra (50 kDa) concentrators (Millipore, Burlington, MA, USA, UFC905024) and the stability and purity of the proteins were determined using SEC-HPLC and SDS-PAGE.

The IOS-1002 molecule was also expressed in CHO cell line and produced and quality tested at a partner CRO site (ProBiogen: https://www.probiogen.de, accessed on 16 August 2024). To generate an isotype control that closely mimics IOS-1002 structure, HLA IgG4 control molecule was constructed as a c- terminal human IgG4 Fc molecule conjugated to the alpha3 domain of HLA-B57:01:01. To abrogate the residual binding of LILR receptors, the alpha3 domain was rationally mutated on putative residues based on in silico models which are predicted to interact with the LILRB2 receptor. The TTI-622 molecule and the antagonistic anti LLILRB1 (BND-22), LILRB2 (1E1, MK-4830) and dual anti LILRB1/LILRB2 (NGM-707) antibodies were generated using the sequences obtained from public sources. Antibodies were purified by HiTrap PrismA protein A based affinity (Cytiva) followed by SEC polishing, if required for >95% monomeric content. The receptor molecules human LILRB1, LILRB2, KIR3DL1 were constructed through ligation of extracellular domains to a c-terminal mouse IgG2aFc with a distal Avi tag (GLNDIFEAQKIEWHE) and expressed transiently in CHO cells. The expressed LILRB1, LILRB2 and KIR3DL1 were further purified by HiTrap PrismA protein A based affinity followed by SEC. The purified LILRB1, LILRB2 and KIR3DL1 were subsequently biotinylated on the c-terminal Avi tag using BirA ligase (Avidity LLC, Aurora, CO, USA, BirA500-RT).

### 2.4. Biophysical Characterization

Purified proteins were analyzed by size exclusion chromatography, using a Thermo Vanquish HPLC with a TSKgel^®^ G3000SWXL SEC Column (Tosoh, Tokyo, Japan, #808541) in PBS with a standard flow rate at 1 ml/min. SDS-PAGE was performed using 4–15% Mini-PROTEAN^®^ TGX Stain-Free gels (BioRad, Hercules, CA, USA, #4568084) in 1× Tris/Glycine/SDS buffer (BioRad, Hercules, CA, USA, #1610772). The thermal shift assay was performed using SYPRO Orange dye (Thermo, Waltham, MA, USA, S6651) according to the manufacturer’s instructions, with a Quant-Studio 5 Real-Time PCR machine (Thermo, Waltham, US). RP-HPLC and subsequent deglycosylated intact mass analysis was performed by the Functional Genomic Centre Zurich (FGCZ). Samples were deglycosylated using PNGase F Immobilized Microspin 5 × 0.2 mg (Genovis, Lund, Sweden, G1-PF6-010) before injection onto a BioResolve-RP-mAb 2.7 μm 2.1 × 150 450 A UPLC column (Waters, Milford, US) on an ACQUITY UPLC (Waters, Milford, MA, USA) and analyzed using TOF MS ES+ on a Synapt G2-Si mass spectrometer (Waters, Milford, MA, USA).

### 2.5. Binding Affinity Quantifications by SPR

Surface plasmon resonance (SPR) technology was utilized for the binding affinity measurements using Biacore T200 instrument (GE Healthcare, Chiacago, IL, USA). For the measurement of equilibrium binding constants biotinylated LILRB1-mFc, LILRB2-Fc and KIR3DL1 proteins were used, produced and biotinylated in-house, immobilized on Series S CAP chip and tested for interaction with IOS-1002 using 5 concentrations at 2-fold differences between 6 and 0.375 μM. The analyses were performed in HBS-EP buffer at 40 μL/min flow rate and at 25 °C with 240 s for association and 360 s for dissociation.

### 2.6. Binding Affinity Quantifications by BLI

Octet based Bio-Layer Interferometry Technology (BLI) was utilized for binding affinity measurements. For the measurement of equilibrium binding constants, the assays were set up such that the C-terminally biotinylated receptors (LILRA1-3 and LILRA5,6) and (LILRB1-5) proteins were immobilized on streptavidin (SAXS) biosensors. The respective biosensors were incubated in increasing concentration of the analytes diluted in PBS and sensorgrams were recorded. Competitive binding to LILRB1 and LILRB2 was performed using SAX biosensors and biotinylated receptors. After capture, the receptors were first blocked with high affinity antibodies to LILRB1 or LILRB2 at 1µM concentration, before the biosensor was placed in IOS-1002 at 2.5 µM concentration. The data analysis, double reference subtraction and quantification of the binding affinities and kinetic parameter were performed using Data Analysis HT 12.0.2.59 software.

### 2.7. Binding EC50 Quantifications by ELISA

Enzyme-Linked Immunosorbent Assay (ELISA) was used for EC_50_ quantification. Streptavidin 96 well plates (Pierce, Waltham, MA, USA, 15500) were coated with biotinylated LILRB1, LILRB2 and KIR3DL1 at 5 μg/mL in PBS. After blocking with 3% BSA in PBS and washing (PBS, 0.01% Tween-20) different concentration series of IOS-1002 and isotype control were added to the wells diluted in buffer (PBS, 0.01% Tween-20). HRP conjugated goat F(ab’)2 Anti-Human IgG, Mouse ads-HRP (SouthernBiotech, Birmingham, AL, USA, 2043-05) was used as secondary antibody. Optical density (OD) values were recorded at 450 nm using Infinite M Nano (Tecan Männedorf, Switzerland). Analysis of data was performed and EC_50_ was calculated with nonlinear fit of XY data-dose/response using GraphPad Prism software (9.4.1).

### 2.8. Cell Lines

HCT116 (BPS Biosciences, San Diego, CA, USA, 60623), H460 (DSMZ, Braunschweig, Germany, ACC 737), MIA PaCa-2 (CLS, Eppelheim, Germany, 300438) and H1703 (ATCC, Manassas, VA, USA, CRL-5889), RPMI-8226 (DSMZ, Braunschweig, Germany, ACC 402). CHO cell expressing human LILRB1, LILRB2 and FcγRI is a stable clone generated by transducing LILRB1 (G&P Biosciences, Santa Clara, CA, USA, LTV2991) and LILRB2 (G&P Biosciences, Santa Clara, CA, USA, LTV2992Z) and nucleofection of CD64 plasmid (GenScript, Piscataway, NJ, USA) to CHO-K1 cell line (Merck, Darmstadt, Germany, 85051005-1VL). H460, H1703, RPMI-8226 were cultured in RPMI1640 GlutaMax media (ThermoFisher, Waltham, MA, USA, 61870044) supplemented with 10% fetal bovine serum (FBS) (ThermoFisher, Waltham, MA, USA, A3840102), 100 U/mL penicillin G and 100 µg/mL streptomycin (ThermoFisher, Waltham, MA, USA, 15070063), MIA PaCa-2 was cultured in DMEM GlutaMax media (ThermoFisher, Waltham, MA, USA, 10566016) supplemented with 10% FBS, 100 U/mL penicillin G and 100 µg/mL streptomycin, HCT116 was cultured in McCoy’s 5A media (ThermoFisher, Waltham, MA, USA, 16600082) supplemented with 10% FBS, 100 U/mL penicillin G and 100 µg/mL streptomycin and 1mg/mL Geneticin (ThermoFisher, Waltham, MA, USA, 10131035), CHO expressing LILRB1 (Ham’s F-12 Nutrient Mix GlutaMAX media (ThermoFisher, Waltham, MA, USA, 31765068) supplemented with 10% FBS, 100 U/mL penicillin G and 100 µg/mL streptomycin, 2 µg/mL Puromycin (Invivogen, Waltham, MA, USA, ant-pr-1)), CHO expressing LILRB2 (Ham’s F-12 Nutrient Mix GlutaMAX media supplemented with 10% FBS, 100 U/mL penicillin G and 100 µg/mL streptomycin, 1 µg/mL Bleomycin (MedChemExpress, Monmouth Junction, NJ, US, HY-17565A)), CHO expressing KIR3DL1 (Ham’s F-12 Nutrient Mix GlutaMAX media supplemented with 10% FBS, 100 U/mL penicillin G and 100 µg/mL streptomycin, 300 µg/mL Hygromycin (AG scientific, San Diego, CA, USA, HY-17565A)), CHO expressing LILRB1/LILRB2/CD64 (Ham’s F-12 Nutrient Mix GlutaMAX media supplemented with 10% FBS, 100 U/mL penicillin G and 100 µg/mL streptomycin, 2 µg/mL Puromycin, 1 µg/mL Bleomycin and 400 µg/mL Hygromycin).

### 2.9. Isolation and Culture of Primary Cells

Peripheral blood samples (anonymized blood donations from healthy adult donors) were purchased from Blood Donation Center Zurich. PBMCs were isolated using density centrifugation with Lymphoprep solution (StemCell Technologies, Vancouver, Canada, 07801). NK cells, T cells and monocytes were isolated from PBMCs using NK cell (17955), T cell (17951) and monocytes (19058) isolation kit (StemCell Technologies Vancouver, Canada), respectively, following the manufacturer’s instructions. NK cells were cultured overnight in NK cell media (Miltenyi Biotech, Bergisch Gladbach, Germany, 130-114-429) with the addition of 500 U/mL of rhIL2 (Miltenyi Biotech, Bergisch Gladbach, Germany, 130-097-746), 50 U/mL of rhIL15 (R&D systems, Minneapolis, MN, USA, 247-ILB-005) and 5% AB human serum (Merck, Darmstadt, Germany, H4522). Following overnight activation, NK cells were resuspended in NK cell media with the addition of 100 U/mL of rhIL2 and 5% AB human serum for follow-up experiments. T cells were cultured in RPMI-1640 growth medium containing 10% FBS and 50 U/mL of rhIL2 for 48h before the experiment. Isolated monocytes were either used immediately or differentiated into macrophages by culture for 5–7 days in ImmunCult-SF macrophage medium (StemCell Technologies, Vancouver, Canada, 19061) in the presence of 50 ng/mL of rhM-CSF (StemCell Technologies, Vancouver, Canada, 78057.1). Monocytes and macrophages were stained with LILRB1 (Biolegend, San Diego, CA, USA, 333708), LILRB2 (Biolegend, San Diego, CA, USA, 338706) and CD64 (Biolegend, San Diego, CA, USA, 305007) antibodies and measured by flow cytometry to assess the expression level of the receptors.

### 2.10. Flow Cytometry Analysis

For surface marker staining, harvested cells were washed once with FACS buffer (PBS containing 2% FBS) and whenever needed, incubated with Human TruStain FcX™ (Biolegend, San Diego, CA, USA, 422302) diluted in FACS buffer (1:20) at room temperature for 10 min. After removing the Human TruStain FcX™, cells were incubated with a cocktail of 2 µg/mL antibodies and DAPI (1:100) diluted in FACS buffer at 4 °C for 20 min. Cells were washed once with FACS buffer and acquired at NovoCyte flow cytometer. The live cell population was first gated based on DAPI negative cells and the expression of receptors in live cells was further analyzed. The positive population of antibody staining was gated based on the cells stained with isotype control antibody.

### 2.11. Cellular Binding Assay

For assessing the binding to CHO cells transduced with individual LILRB1, LILRB2 and KIR3DL1 receptor, CHO cells were incubated at 37 °C for 2 h with multiple concentrations of unconjugated compounds. After washing with FACS buffer, cells were incubated at 4 °C for 20 min with a cocktail of APC-conjugated goat anti-human IgG antibody (Jackson ImmunoReseach, West Grove, PA, USA, 109-135-098) and DAPI (Miltenyi Biotech, Bergisch Gladbach, Germany, 130-111-570). Cells were washed once with FACS buffer and acquired at NovoCyte flow cytometer (Agilent, Santa Clara, CA, USA). Assessment of binding to CHO cells co-expressing LILRB1, LILRB2 and CD64 was performed with compounds conjugated with AlexaFluor488 using Protein Labeling Kit (Invitrogen, Waltham, MA, USA, A10235). Cells were incubated at 37 °C for 2 h with multiple concentrations of AlexaFluor488 conjugated compounds. Cells were washed once in PBS buffer after incubation and stained with Zombie Violet (Biolegend, San Diego, CA, USA, 301829) at room temperature for 15 min. Cells were washed once with FACS buffer and acquired at NovoCyte flow cytometer. Assessment of binding of IOS-1002 to human primary monocytes and macrophages was performed in isolated monocytes and macrophages derived from the same donors from whole blood of healthy donors. Cells were incubated at 37 °C for 2 h with multiple concentrations of unconjugated compounds. Following the blocking of the Fc receptors with Human TruStain FcX™ (Biolegend, San Diego, CA, USA, 422302) at 4 °C for 10 min, cells were incubated at 4 °C for 20 min with a cocktail of 2 µg/mL anti-G4S linker antibody (Cell Signaling Technology, Danvers, MA, USA, 50515) and DAPI. Cells were washed once with FACS buffer and acquired at NovoCyte flow cytometer. The MFI value of binding was acquired from the live cell/DAPI negative population.

### 2.12. Competitive Binding Assay

Assessment of competitive binding to LILRB1, LILRB2 and CD64 in human primary monocytes was performed on isolated monocytes from whole blood of healthy donors. Fifty thousand monocytes per well were seeded in a 96 well plate and were incubated with 100 µg/mL isotype, anti-LILRB1 (BND-22), anti-LILRB2 (MK-4830), anti-LILRB1/LILRB2 (NGM707), anti-CD64 or a combination of anti-LILRB1/LILRB2 and anti-CD64 antibodies in IgG1 Fc null backbone at 37 °C for 30 min. Subsequently, unconjugated IOS-1002 in IgG4 format was added on top directly to a final concentration at 1282 nM and incubated at 37 °C for 2 h. Following blocking the Fc receptors with Human TruStain FcX™ at 4 °C for 10 min, cells were incubated at 4 °C for 20 min with a cocktail of 2 µg/mL anti-G4S linker antibody and DAPI. Cells were washed once with FACS buffer and acquired at NovoCyte flow cytometer. The MFI value of binding was acquired from the live cell/DAPI negative population.

### 2.13. SHP1/2 Phosphorylation Analysis with Simple Western

For analysis of ITIM signaling, JESS Simple Western system (ProteinSimple, San Jose, CA, USA) was used. Monocyte-derived macrophages were treated with the indicated compounds (20 µg/mL; IgG4 isotype (Biolegend, San Diego, CA, USA, 403702) or IOS-1002) on day 1 and day 5 post isolation. On day 6, cells were collected for protein extraction by gentle scraping on ice. Subsequently, scraped macrophages were washed twice in ice-cold PBS and lysed by incubating in RIPA lysis buffer (ThermoFisher Scientific, Waltham, MA, USA, 89900), which contained completed™ Protease Inhibitor Cocktail (Roche, Basel Switzerland, 11697498001), Phosphatase inhibitor PhosSTOP (Roche, Basel, Switzerland, 4906845001), Phosphatase Inhibitor Cocktail 2 and 3 (Sigma-Aldrich, St. Louis, MO, USA, P5726 and P0044) for 30 min on ice. Protein concentration was quantified using Pierce BCA Protein Assay Kit (ThermoFisher Scientific, Waltham, MA, USA, 23225) according to manufacturer’s instructions.

Antibodies against p-SHP2 (R&D systems, Minneapolis, MN, USA, AF3790-SP), SHP2 (Santa Cruz, Dallas, TX, USA, sc-7384), p-SHP1, SHP1 (both Cell Signaling, Danvers, MA, USA, 8849; 3759S, respectively) were used. Secondary antibodies were provided with the JESS detection modules (Bio-Techne, Minneapolis, MN, USA). As loading control, the Total Protein Detection Module (Bio-Techne, Minneapolis, MN, USA, DM-TP01) or the specific total protein were used. Changes in levels of protein expression upon drug administration are shown as the peak area of the phosphorylated protein over the peak area of the total protein relative to isotype control.

### 2.14. Generation of MDSCs

For differentiation of MDSCs, CD14^+^ monocytes were isolated from human PBMCs (*n* = 3) using immunomagnetic isolation (EasySep Human CD14 Positive Selection Kit II, StemCell, Vancouver, Canada, 17858), resuspended in RPMI-1640 (RPMI-1640, 10% heat inactivated FBS, 100 U/mL penicillin, 100 µg/mL streptomycin and 2mM L-glutamine, 50 µM β-mercaptoethanol, AQ-171) supplemented with recombinant human GM-CSF (20 ng/mL, Peprotech, Waltham, MA, USA, 111930) and recombinant human IL-6 (20 ng/mL, Peprotech, Waltham, MA, USA, 031916) in 96-well flat-bottom plates and cultured for six days at 37 °C and 5% CO_2_. Compounds were added on day 0 and replenished on day 3. On day 6, adherent and non-adherent cells were recovered and cells were stained for viability, CD33 (ThermoFisher, Waltham, MA, USA, 17-0338-42), CD15 (Biolegend, San Diego, CA, USA, 323031), CD11b (ThermoFisher, Waltham, MA, USA, 11-0118-42), CD14 (Biolegend, San Diego, CA, USA, 301838) and HLA-DR (ThermoFisher, Waltham, MA, USA, 45-9956-42) to assess MDSC viability and phenotype by flow cytometry. Data show the mean (+SEM, *n* = 3) proportion of MDSCs (CD11b^+^CD14^+^CD15-CD33^+^HLA-DR^−^) gated from viable cells.

### 2.15. Macrophage Polarization Assay

Primary monocyte-derived macrophages were treated with the specified compounds (20 µg/mL, IgG4 isotype (Biolegend, San Diego, CA, USA, 403702) or IOS-1002/a-LILRB2 ab) at day 1 and 5 post-isolation. On the following day, macrophages were collected and polarization analysis was performed with flow cytometry by assessment of CD23 (Biolegend, San Diego, CA, USA, 338516), CD206 (Biolegend, San Diego, CA, USA, 321104) and CD209 (Biolegend, San Diego, CA, USA, 330118) expression. Each polarization reaction was performed in technical duplicates.

### 2.16. Macrophage Phagocytosis

For phagocytosis of MIA PaCa-2 and RPMI-8226 cells, primary monocyte-derived macrophages were pre-treated with compounds also at day 1 post isolation. For phagocytosis of H460 cells, primary monocyte-derived macrophages were activated with 200 ng/mL of IFNγ (Peprotech, Waltham, MA, USA, 300-02) for 48 h at day 5–6 post-isolation and boosted with 40 ng/mL of LPS (Sigma Aldrich, L6529-1MG) 1h before phagocytosis experiment. On the day of the experiment, macrophages were treated with compounds as indicated; cancer cells were labeled with CellTrace CFSE (ThermoFisher, Waltham, MA, USA, C34554) and pHrodo iFL Red STP Ester (Invitrogen, Waltham, MA, USA, P36010) according to manufacturer’s instructions and added to the wells with E:T ratio of 1:2. Treatments were performed using 20 µg/mL of compounds unless differently indicated and commercial isotype controls used in the experiments were: IgG4 isotype (Biolegend, San Diego, CA, USA, 403702; Abinvivo, Shanghai, China, B669701; Lucerna Chem, Luzern, Switzerland, ICH2257S228P), IgG1^N297G^ Isotype (Abinvivo, Shanghai, China, B22890601); IgG1^LALAPG^ Isotype (Abinvivo, Shanghai, China, B422203). Co-cultures were monitored with the S3 or SX5 IncuCyte live cell imaging system, 4 non-adjacent 10× objective fields per well were imaged every 2 h and processed for analysis with Incucyte software v2020C/2022a. Quantification of phagocytic events was based on segmentation of the red signal deriving from the pHrodo in red objects. The green signal generated by CFSE stained cancer cells was used to define green objects and used to exclude background red signal from cancer cells. Using phase contrast, all cells present within the imaged field were segmented into objects to determine the cell density, calculated as percentage of confluence. In the reported data, the phagocytosis values represent the red integrated intensity per well normalized over the confluence per well (Red Calibrated Unit × µm^2^/Well/percentage of confluency; relative integrated intensity pHrodo). Analysis of phagocytosis was conducted at the endpoint (when it reached its maximum and the kinetic flattened toward a plateau). To standardize the donor-to-donor variability, values were normalized to the highest phagocytosis score measured across technical replicates per donor (normalized phagocytosis). To quantify the EC_50_ and to estimate the potency of IOS-1002 in phagocytosis assays, dose-response curves were built by calculating the area under the curve (AUC) of the phagocytosis over time for each specific dose; this approach enables including time as a factor in potency estimation.

### 2.17. Cancer Cell and T Cell/NK Cell Co-Culture Assay

For the co-culture assays, isolated human T and NK cells were seeded in flat bottom 96 well plates, treated with indicated compounds (20 µg/mL of IgG4 isotype (Biolegend, San Diego, CA, USA, 403702) or IOS-1002, 10 µg/mL of PD-1 antibody (BioXcell, Lebanon, NH, USA, SIM0010)) and mixed with cancer cells (2000 cells). The number of seeded immune cells was determined by the chosen E (effector): T (target) cell ratio, which is E:T of 1:5 for T cells experiments and E:T of 2:1 for NK cells. Killing of cancer cell was monitored over time by live cell imaging. Live-cell imaging was performed using the Incucyte S3 or SX5 Live-Cell Analysis System (Sartorius) and Incucyte software v2020C/v2022a. Employing a 10× objective, 4 non-adjacent fields per well were imaged every 2–4 h and processed for analysis. Cancer cells were stained with CellTrace™ CFSE (ThermoFisher, Waltham, MA, USA, C34554) according to the manufacturer’s instructions and the CFSE signal of each cell was segmented in green objects. Every green object was counted as cancer cells. Dead cells were identified by segmentation in blue objects the blue signal produced by the binding of the Cytotox NIR (Sartorius, Göttingen, Germany, 4846) to the DNA. Cancer cell death was detected by colocalization of a green object with a blue one. Analysis of T cells experiments was performed by using the “Incucyte Cell-by-Cell Analysis Software Module (9600-0031)” (Sartorius, Göttingen, Germany). In brief, cells were divided in single cells using with a phase contrast mask, cancer cells and dead cells were marked by CFSE and Cytotox signal respectively and different cell populations were counted over time. In the reported data, the proliferation values represent the fold changes of the alive T cells number over timepoint 0; the percentage of cancer cell death represents the relative number of dead cancer cells per well over total cancer cells per well.

### 2.18. PBMCs and Cancer Cells Co-Culture TNFa Secretion Assay

Human primary PBMCs were seeded in U-bottom 96-well plates, treated with compounds (25 µg/mL of IgG4 isotype (Abinvivo, Shanghai, China, B669701; Lucerna Chem, Luzern, Switzerland, ICH2257S228P) or IOS-1002, 10 µg/mL of PD-1 antibody (BioXcell, Lebanon, NH, USA, SIM0010)) and co-cultured with H1703 cancer cells for 48 h in E:T ratio of 1:3. Subsequently, cell supernatants were harvested and processed for TNFa analysis. The culture medium was composed by RPMI1640 GlutaMax media supplemented with 10% FBS, 100U/mL penicillin G, 100 µg/mL streptomycin, 50U/mL of rhIL2, 1 µg/mL of anti-CD3 (Invitrogen, Waltham, MA, USA, MA1-10175) and 1 µg/mL of anti-CD28 (Invitrogen, Waltham, MA, USA, MA1-10166) antibodies. TNFa quantification from the cell supernatants was performed with ELISA MAX Deluxe Set Human TNFa (Biolegend, San Diego, CA, USA, 430215) according to manufacturer’s instructions.

### 2.19. Ex Vivo Patient Tumor Study

Ex vivo patient tumor study was performed using Crown Bioscience proprietary assay. In short, the resected primary human material was disaggregated into multicellular micro-tissues (tumoroids) and embedded in a protein-rich hydrogel. The embedded tumoroids are exposed to test articles at various doses in a 384-well format for 6–8 days. The effect of test articles is estimated by observing the changes in total tumoroid area as measured by the company’s proprietary automated high-content imaging analysis platform. Using object segmentation of the stained cell material (DAPI for nuclei, actin for cell clusters) the cell content of each well is quantified. An image stack is acquired for all wells in the 384 well plate and analyzed per section to obtain data representative of the 3D culture. The pixel area of individual cell/tumor clusters is measured and the total tumoroid size is the sum of all objects larger than the predefined cut-off present in a well. Staphylococcal enterotoxin A (SEA) strongly activates human lymphocytes and is used in the assay as a positive control for the tumoroids infiltrated by the immune cells and potentially responsive to immunotherapy.

### 2.20. Statistics

All statistical analyses were performed using GraphPad Prism, version 9.4.1. Detailed statistical methods are provided in the respective figure legends. For all analyses, a *p*-value of <0.05 was considered significant.

## 3. Results

**Figure 1:**
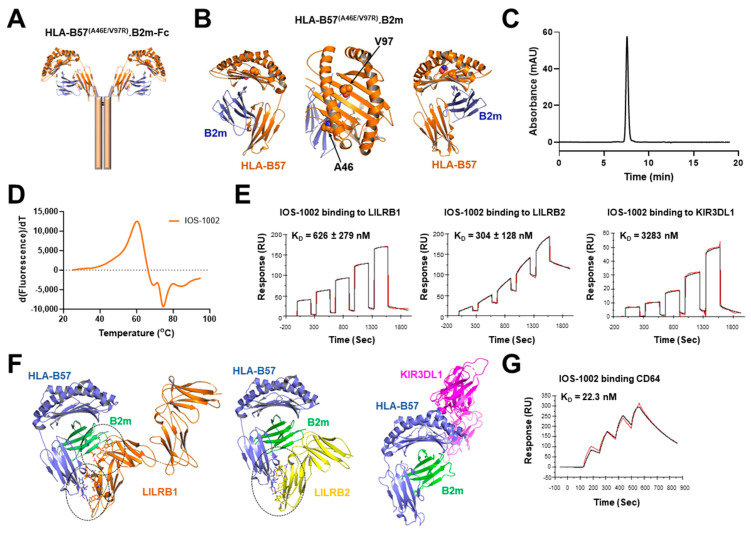
Structure, expression and receptor binding characteristics of IOS-1002. (**A**) Schematic representation of the IOS-1002 molecule constructed through the ligation of HLA-B57^(A46E/V97R)^ on N-terminus of human IgG4 Fc domain. (**B**) The topological structure of HLA-B57:01:01 including the B2m molecule. Mutation site residues A46 and V97 highlighted as spheres (PDB: 5VUF). (**C**) SEC-HPLC profile of purified IOS-1002. (**D**) Thermal unfolding profile of IOS-1002, determined by DSF. (**E**) Quantification of the binding affinities of IOS-1002 to LILRB1 (*n* = 4), LILRB2 (*n* = 5) and KIR3DL1 (*n* = 1) surface receptors determined by SPR. Red line represents raw data and black line represents the fit of 1:1 binding. RU: response units; K_D_: binding constant represented as mean ± standard deviation. (**F**) The topological structure of the HLA-B57:01:01 interaction site generated by superimposing the HLA-B57 structure (PDB: 2HJK) onto LILRB1/HLA-G and LILRB2/HLA-G. The residues lining the binding interfaces between HLA-B57-B2m:LILRB1 and HLA-B57-B2m:LILRB2 are highlighted under the dashed circles and displayed as sticks. The crystal structure of HLA-B57:01 and KIR3DL1 allotype 015 (PDB: 5B39), which describes a separate epitope on the HLA-B57 α1-helix, incorporating residues 77–83, known as the Bw4 motif. Structural images generated using PyMOL. (**G**) Quantification of the binding affinity of IOS-1002 to FcγRI determined by SPR (*n* = 1). The specified *n* indicates the number of independent experiments.

The HLA-B57^(A46E/V97R)^-IgG4_Fc (IOS-1002) fusion protein construct was rationally designed so that the cell surface exposed extracellular domains (α1-α3) of HLA-B57:01:01 were N-terminally linked to a human IgG4 Fc backbone to obtain a chimeric homodimer of the extracellular HLA-B57 domain (Figure 1A). Based on in silico assessments for protein stability and expression analysis, two amino acids at position 46 and 97 (based on uniport numbering) of the extracellular HLA-B57 were replaced by the respective substituted amino acids A46E, V97R (Figure 1B). For recombinant production, the CHO cells were transiently co-transfected with HLA-B57-IgG4_Fc variants and human B2m (Supplemental Figure S1A). The optimized variant (HLA-B57^(A46E/V97R)^.B2m-IgG4_Fc) had a higher number of viable cells that correlated with significantly higher protein expression titers at different HLA:B2m ratios when compared to the parental (HLA-B57-IgG4_Fc) construct (Supplemental Figure S1A). In addition, purification and stability profiles for the optimized variant were superior as compared to the wild type (HLA-B57.B2m-IgG4_Fc) construct (Figure 1C and Supplemental Figure S1B). When applying thermal stress to this variant the first unfolding transition was observed by DSF at 60 °C, similar to what is expected for an IgG4 CH2 domain [19] (Figure 1D).

To identify receptors as binding partners for IOS-1002, systematic interaction studies with human LILRs (LILRB1-5 and LILRA1-6) and human KIR3DL1 receptor were performed. No binding of IOS-1002 was detected to members of the LILRA (LILRA1-6) and LILRB3-5 receptor family as measured by BLI (Supplemental Figure S1C). However, using two orthogonal methods, we were able to quantify IOS-1002 binding to LILRB1, LILRB2 and KIR3DL1 in a dose-dependent manner and determined binding K_DS_ and EC_50_ values by SPR and ELISA, respectively (Figure 1E and Supplemental Figure S1D). For SPR, C-terminally biotinylated LILRB1, LILRB2 and KIR3DL1 human proteins with either a C-terminal His or mouse Fc tag were immobilized on a streptavidin surface and used in a similar way. Representative SPR sensorgrams are shown in Figure 1E, with data selected only if the fitting allowed for accurate kinetic values to be calculated. After averaging multiple experiments, K_D_ ranges for IOS-1002 were calculated using 1:1 fits as 626 nM (St. Dev. 279 nM) for LILRB1 and 304 nM (St. Dev. 128 nM) for LILRB2. For KIR3DL1, IOS-1002 binding was in the low micromolar range with a K_D_ value of 3283 nM calculated with a 1:1 fit (Figure 1E), while the averaging of steady-state fits gave the K_D_ as 7.48 µM (St. Dev. 1.120 µM; Figure 1E). Additionally, binding to all three receptors was confirmed in CHO cells transduced with each receptor (Supplemental Figure S1F). Identification of the contact residues between IOS-1002 and LILRB1 or LILRB2 receptors was performed by superimposing the HLA-B57 structure (PDB: 2HJK) onto LILRB1/HLA-G and LILRB2/HLA-G (PDB: 6AEE and 2DYP), respectively. The KIR3DL1 epitope was known to be distinct as can be seen for the crystal structure of HLA-B57:01 and KIR3DL1:015 (PDB 5B39) (Figure 1F).

Since the Fc region of IOS-1002 can interact with FcγRs, we also evaluated the binding affinities of IOS-1002 to CD64 (FcγRI) and CD32a (FcγRIIa)/CD32b (FcγRIIb) receptors, which are reported to have high to medium affinity, respectively. As shown in Figure 1G (and Supplemental Figure S1G), the binding affinity of IOS-1002 is similar to reported values for the Fc portion of IgG4 antibodies [20], indicating that HLA fusion onto the Fc did not change the binding potential to Fc target receptors. Comparisons of affinities of IOS-1002 to LILRB1, LILRB2, KIR3DL1 and FcγRs show higher affinity for CD64 (Figure 1E,G).

**Figure 2:**
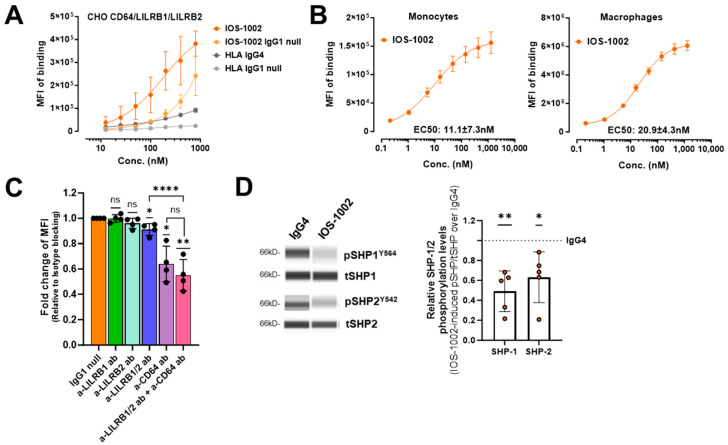
IOS-1002 binds to target receptors on human primary cells and inhibits the associated downstream signaling. (**A**) CHO cells were transduced with LILRB1, LILRB2 and CD64 FcγRI and interaction of AF488 labeled molecules was measured by flow cytometry (*n* = 2). Mean ± standard deviation is presented. MFI: median of fluorescence intensity. (**B**) Dose-dependent binding of IOS-1002 on human primary monocytes and the monocyte-derived macrophages isolated from PBMCs (*n* = 4). Mean ± standard deviation is presented. MFI: mean of fluorescence intensity. The non-linear regression curve and EC_50_ (95% Confidential Interval) were calculated using the model agonist vs. response variable slope (four parameters) in A and B. (**C**) Competition between IOS-1002, anti-LILRB1, anti-LILRB2, dual anti-LILRB1/2 and anti-CD64 antibody for cell surface epitopes on monocytes. Fold change of background-subtracted MFI relative to the cells pre-treated with IgG1 null antibody is presented, (*n* = 4). Mean ± standard deviation is presented. Statistical analysis of various conditions against IgG1 null control was performed using one-sample *t*-test (hypothetical mean = 1) and pre-treatment of combined dual anti-LILRB1/2 and anti-CD64 antibodies against anti-LILRB1/2 or anti-CD64 antibodies was analyzed by one-way ANOVA with Bonferroni multiple comparisons test. (**D**) Simple Western analysis showing expression and phosphorylation of ITIM-associated phosphatases, SHP-1 and SHP-2 in human primary monocytes-derived macrophages (*n* = 5). Quantification of phosphorylation over total protein relative to isotype control is presented in the graph on the right. Mean ± standard deviation is presented. Stars indicate the statistical significance against IgG4 control (one-sample *t*-test, hypothetical mean = 1). * *p* < 0.05; ** *p* < 0.01; **** *p* < 0.0001. ns, non-significant. In (**A**) *n* indicates the number of independent experiments, in (**B**–**D**) *n* indicates the number of independent donors.

To understand the contribution of each individual receptor to IOS-1002 binding, we transduced CHO cells with LILRB1, LILRB2 and CD64, mimicking simultaneous expression on myeloid cells. Assessment of IOS-1002 binding to these CHO cells showed increased overall binding for cells expressing LILRB1 and LILRB2 target receptors in the presence of CD64 (Figure 2A). When calculating the collective binding affinity to all receptors, a synergistic effect was observed between Fc and HLA binding to target receptors, which hinted toward generation of an avidity effect in the presence of all three receptors. This was not observed for antibodies with high affinity to LILRB1 and LILRB2 with active IgG4 Fc, as binding affinity seems to be mainly driven by LILRBs, while Fc binding has less contribution (Supplemental Figure S2A). Since monocytes and macrophages are the main immune cell population with simultaneous expression of LILRB1, LILRB2 and CD64 receptors (Supplemental Figure S2B), we assessed binding of IOS-1002 to this population and determined low nanomolar EC_50_ values (Figure 2B). Next, we determined the binding specificity of IOS-1002 to all three receptors and tested a diverse range of antibody clones, which compete for the same epitope as IOS-1002 to LILRB1 and LILRB2 receptors (Supplemental Figure S2C). Using these antibodies, we blocked each of the target receptors with and without the combination of a specific CD64 antibody followed by labelled IOS-1002 stain. Results demonstrated that the single blockade of either LILRB1, LILRB2 or simultaneous LILRB1/LILRB2 antibodies reduced the binding of IOS-1002 to monocytes whilst the combination of LILRB1, LILRB2 and CD64 was additive and significantly reduced the binding of IOS-1002 (Figure 2C). We were not able to completely block the binding, which suggests additional binding to other FcγRs subtypes expressed on monocytes. Similarly to results observed in CHO transduced cells, the strongest binding was shown to be derived from CD64. Next, we evaluated if the binding of IOS-1002 to monocytes affects the ITIM signaling downstream of LILRB1 and LILRB2 receptors, employing Simple Western Technology (Figure 2D). Treatment of monocytes with IOS-1002 in culture during differentiation to macrophages reduced receptor-mediated activation of the ITIM-related downstream signaling, as demonstrated by decreased phosphorylation levels of SHP-1 and SHP-2 tyrosine phosphatases (Figure 2D and Supplemental Figure S2D). Taken together, these results demonstrate that IOS-1002 is binding to LILRB1, LILRB2 and FcγRs (CD64 was mainly tested here due to highest binding affinity) and this binding impacts downstream signaling in cells expressing the receptors.

**Figure 3:**
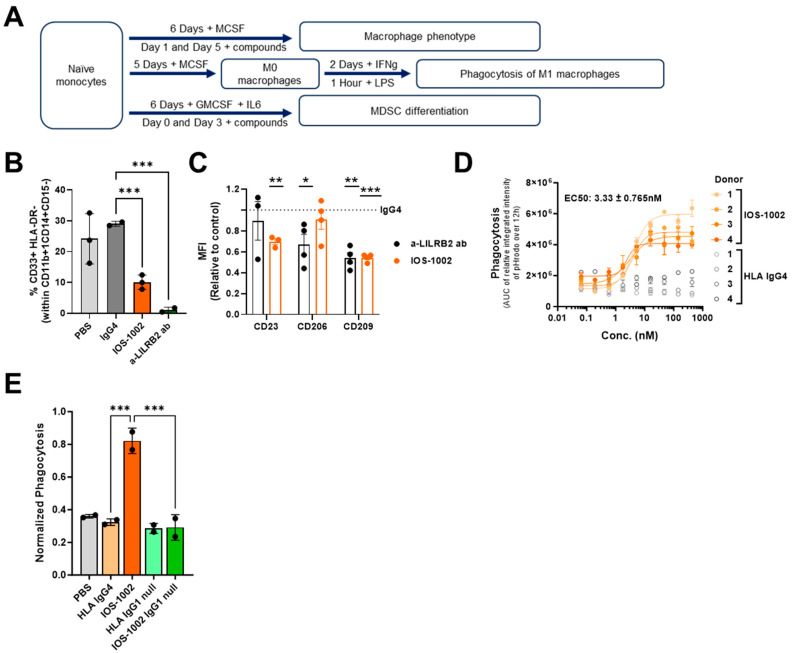
IOS-1002 affects the differentiation of monocytes toward MDSCs and enhances phagocytosis of monocyte-derived macrophages. (**A**) Scheme of different monocyte-derived immune cell-based assays performed. (**B**,**C**) The effect of IOS-1002 on the differentiation potential of monocytes toward MDSCs (*n* = 3) (**B**) and M2 macrophages (*n* = 4) (**C**) is presented and compared with anti-LILRB2 antibody. Mean ± standard deviation is presented. In C, stars indicate the statistical significance toward IgG4 control. (**D**) Macrophage phagocytosis in the presence of different concentrations of IOS-1002 toward H460 (NSCLC cell line) (*n* = 4). Mean ± standard deviation of 3 technical replicates is presented. A 4P-L curve was interpolated for quantification of the EC_50_. (**E**) Macrophage phagocytosis in the presence of IOS-1002 on different Fc backbones toward H460 cell line (*n* = 2). Mean ± standard deviation is presented. Statistical analysis was performed using one-way ANOVA and Dunnett’s multiple comparisons test. Unless mentioned otherwise, all indicated compounds were used at a concentration of 20ug/mL. * *p*  <  0.05, ** *p*  <  0.01, *** *p*  <  0.001. The specified *n* indicates the number of independent donors.

To better understand the effect of IOS-1002 treatment on different myeloid cell populations, we performed diverse immune cell-based assays that included the use of human primary monocytes. Since monocytes can differentiate into MDSCs which are active in tumor maintenance and progression [21], we assessed the polarization of monocytes towards MDSCs in the presence of IOS-1002 (Figure 3A). Results demonstrate that IOS-1002 significantly blocked the polarization of monocytes to monocytic MDSCs (Mo-MDSC) (Figure 3B), without influencing the viability of these cells (Supplemental Figure S3A). A similar effect was observed for a LILRB2 antagonistic antibody, confirming that this effect is related to LILRB2 blockade (Figure 3B).

Macrophages are the most abundant cell population in tumors and express high levels of LILRB1 and LILRB2 receptors [22]. To assess phenotypic changes in primary human macrophages upon IOS-1002 treatment, we treated monocytes on days 1 and 6 of the differentiation period (Figure 3A) and subsequently analyzed phenotypic changes of M2-specific macrophage surface markers. Results demonstrated a decrease in M2-associated surface markers in the presence of IOS-1002 and a LILRB2 blocking antibody, which was not observed with the isotype control (Figure 3C).

Next, we sought to explore whether the ability of IOS-1002 to reverse the effect on markers of alternative macrophages resulted in increased anti-tumor phagocytic activity. For this, we polarized macrophages to M1 classical macrophages (Figure 3A), as this cell type has increased levels of CD64 ([23] and in-house data). Results demonstrated that IOS-1002 increased macrophage phagocytosis in a dose-dependent manner, with a calculated EC_50_ of 3.33nM (Figure 3D and Supplemental Figure S3B). Higher levels of phagocytosis were observed compared to controls including a LILRB1, LILRB2 and a dual LILRB1/LILRB2 antagonistic antibody (Supplemental Figure S3C). Similar results were obtained from undifferentiated M0 macrophages against multiple cancer cell lines, where the level of phagocytosis was higher upon IOS-1002 treatment in comparison to blockade of the CD47/SIRPa “Don’t eat me signal” axis using a SIRPa ligand-Fc fusion protein (Supplemental Figure S3D).

FcγRs have been shown to exert a profound impact on antibody function and in vivo efficacy. Fc endows IgG antibodies with effector functions, which include antibody dependent-cellular cytotoxicity (ADCC), complement-dependent cytotoxicity (CDC), antibody-dependent cellular phagocytosis (ADCP), induction of cytokines/chemokines and endocytosis of opsonized targets [24]. Since high affinity-dual LILRB1/LILRB2 blocking antibody was unable to induce macrophage phagocytosis, we sought to understand the impact of IOS-1002 binding to FcγRs on induction of macrophage phagocytosis. For this, we used IOS-1002 on an inactive IgG format (IgG1 LALAPG) silenced for binding to FcγRs. The results demonstrate that loss of binding to FcγRs completely abrogated the activity of IOS-1002 on macrophage effector function (Figure 3E). This data clearly indicates that engaging LILRB receptors and FcγRs on macrophages with IOS-1002 is an effective strategy of macrophage activity as measured by the increase of phagocytosis against cancer cells. Moreover, an effect of IOS-1002 beyond LILRBs’ blockade is supported.

**Figure 4:**
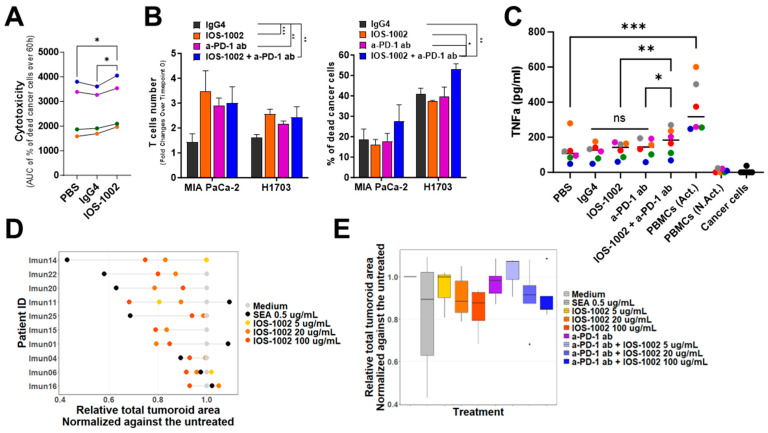
IOS-1002 activates T and NK cells and demonstrates efficacy in ex vivo patient samples. (**A**) Isolated human primary NK cells were incubated with HCT116 colon cancer cell line in a cell-cell contact manner and the percentage of cancer cell killing was measured for 60 h. Area under the curve (AUC) of the percent cytotoxicity over time was calculated and is represented in the graph (*n* = 4). Different colors represent independent donors. Statistical analysis was performed using RM one-way ANOVA with Dunnett’s multiple comparisons test. (**B**) Non-activated T cells were incubated with MIA PaCa-2 (pancreatic carcinoma, *n* = 2) and H1703 (NSCLC, *n* = 2) cancer cell lines in a cell-cell contact manner and co-cultures were monitored for 72 h; left, the T cells number at endpoint (72 h) is presented in fold changes over timepoint 0; right, the number of dead cancer cells at 72 h, expressed in percentage, is represented. Mean ± standard deviation is shown. Statistical analysis was performed using two-way ANOVA with Dunnet’s multiple comparisons. (**C**) TNFa levels in cell supernatant of PBMCs incubated with H1703 in a cell-cell contact manner for 48 h. TNFa concentration (pg/mL) for each individual donor is represented (*n* = 6). Every donor is color-coded throughout the treatments. Statistical analysis was performed using RM one-way ANOVA with Dunnett’s multiple comparisons test. Paired *t*-test analysis was used to compare activated PBMCs monoculture and PBS control co-culture. Act.: Activated, N. Act.: Non-activated. (**D**,**E**) Relative total tumoroid area normalized against the untreated sample and shown for individual patient samples (**D**) and in total cohort (**E**) upon different treatments. Each reported measurement is a median of up to 8 technical replicates. Imun15 sample has no SEA control recorded due to the technical error in the experiment. * *p*  <  0.05, ** *p*  <  0.01, *** *p*  <  0.001. ns, non-significant. The specified *n* indicates the number of independent donors.

NK cells are an important part of the tumor microenvironment (TME) due to their capacity to eliminate malignant cells that may otherwise evade the adaptive immunity through HLA downregulation [25]. The expression of both LILRB1 and KIR3DL1 receptors by NK cells varies between individuals [26]. As IOS-1002 binds to LILRB1 and KIR3DL1 receptors, we investigated the impact of IOS-1002 on NK cytotoxic activity. Results demonstrate that IOS-1002 increases the cytotoxic potential of NK cells against NSCLC cancer cell lines (Figure 4A). In addition to NK cells, a subset of T cells expresses LILRB1 [12] and LILRB1 blockade was shown to enhance bispecific T cell engager antibody-induced tumor cell killing by effector CD8^+^ T cells [27]. Single-cell RNA-seq data has shown that LILRB1 and PD-1 receptors are expressed by distinct CD8^+^ T cell subsets in tumors [27]. This evidence suggests that the combination of LILRB1 and PD-1 blockade may be required to maximize tumoricidal activity of CD8^+^ T cells. To address this, a combination of IOS-1002 plus an anti-PD-1 antibody was assessed on resting CD3 T cells, co-cultured with MIA PaCa-2 or H1703 cancer cell lines (Supplemental Figure S4). Co-cultures were treated with compounds and monitored using the IncuCyte live-cell imaging system for 72 h. Results demonstrate that T cell proliferation increased equally among treatment of IOS-1002, anti-PD-1 antibody or combination and not in the IgG4 control group (Supplemental Figure S4), the cytotoxic potential of T cells was increased upon combination of IOS-1002 with anti-PD-1 and not in monotherapy or IgG4 control groups (Figure 4B).

To use a system that more closely resembles the entire immune system consisting of myeloid and lymphoid cells, we used human primary PBMCs as a source of effector cells. We measured the amount of TNFa, as a pro-inflammatory cytokine [28,29], released in the supernatant when co-cultured with H1703 cancer cells. Quantification of TNFa demonstrated that neither cancer cells, nor non-activated PBMCs, produced a significant amount of TNFa (Figure 4C). Upon T cell activation, PBMCs secreted elevated levels of TNFa, ranging from 247.66 to 600.397 pg/mL, indicating a wide donor-to-donor variability. In this system, IOS-1002 and PD-1 alone were unable to induce a significant rise of TNFa secretion. When combined with IOS-1002, PD-1 treatment resulted in a significant increase of TNFa levels in the supernatants (Figure 4C). On average, co-cultures treated with a combination of IOS-1002 and anti PD-1 antibody showed 50% higher levels of TNFa compared to isotype control, 34% more than IOS-1002 and 24% more than anti PD-1 antibody-treated co-cultures (Figure 4C).

Rodents do not express LILRB proteins limiting their usefulness as a model for pre-clinical study of compounds targeting LILRBs [12]. To overcome this limitation and to explore the activity of IOS-1002 in experimental systems of increased translational relevance, we next examined the anti-tumor efficacy of IOS-1002 in 3D ex vivo patient tissue samples (Figure 4D,E), where composition and phenotype of cells more closely reflect the state of the actual TME. For this purpose, fresh human tumor material was acquired post-surgical resection from ten NSCLC patients who were either newly diagnosed or in the last treatment cycle (chemotherapy/radiation) had passed more than 6 months prior to resection. Samples were treated with three different concentrations of IOS-1002. Based on the availability of material, a subset of samples was also treated with anti PD-1 antibody alone and in combination with IOS-1002. As a positive control, SEA activity was interpreted as an indication of overall immunotherapy responsiveness of a given specimen. Results indicate an anti-tumor effect of IOS-1002 at various doses in six out of ten specimens (Figure 4D). Among the four specimens where no IOS-1002 effect was observed, three were deemed generally unresponsive to immunotherapy based on the absence of an SEA effect as positive control. Among the six tumors where IOS-1002 showed anti-tumor activity, one is deemed unresponsive to immunotherapy based on the absence of SEA effect (Figure 4D). Combination of IOS-1002 and anti PD-1 treatment increases efficacy over anti PD-1 antibody monotherapy (Figure 4E). Overall, the study outcome confirmed the IOS-1002 anti-tumor efficacy in a 3D experimental system with ex vivo tumor patient material.

## 4. Discussion

The presence of specific HLA alleles in the human population are associated with the development of autoimmune disease and viral control [10]. We have previously demonstrated that dimers of HLA-B27 open formats could induce the activation of diverse sets of immune cells and that this activation was correlated with the development of Spondylarthritis [3,30] (SpA). Interestingly HLA-B27 is not only associated with SpA but is also a protective allele for HIV and HCV [5]. The mechanisms of the dual role of HLA-B27 in autoimmunity and viral protection are not yet well understood, however interaction to the LILRB2 receptor has been associated as part of the mode of action for both mechanisms [8,9,31].

Since HLA-B27 is not a druggable candidate due to its intrinsic biochemical properties that lead to multimerization in part due to an additional cysteine in position 67 [32], we sought to assess other HLA candidates based on their association to autoimmune diseases and viral protection [33]. To address this, we performed a systematic analysis of different HLA open formats in an Fc-fusion format that could mimic the dimerization of HLA-B27 and increase the avidity of an HLA open format to LILRB and KIR receptors. The lead molecule identified by this approach is an open format variant of HLA-B57:01:01, associated with the development of psoriasis and protection against HIV and HCV [5,33]. HLA-B57:01:01 has a similar profile as HLA-B27 by binding to LILRB1, LILRB2 and KIR3DL1 receptors, both LILRB1 and LILRB2 checkpoint receptors are of wide clinical interest in cancer immunotherapy [34,35]. To improve the stability and manufacturability of HLA-B57:01:01 we substituted two amino acids A46E and V97R, which are conserved residues shared among several other HLA molecules. These specific mutations indeed increased protein stability, CHO cell viability and higher production titers without disrupting receptor interaction, this final lead candidate was termed IOS-1002.

Using in silico analysis, the IOS-1002 epitope binding regions on LILRB1 and LILRB2 receptors were determined by superimposing HLA-B57, LILRB1 and LILRB2 crystal structures. We identified that the IOS-1002 epitope overlaps with the HLA-G binding regions on LILRB1 and LILRB2 [36]. In addition, IOS-1002 competes with different clinical-stage anti-LILRB1 and anti-LILRB2 antibodies for the same epitope as shown by BLI and cell-based assays using monocytes (expressing high levels of LILRB1 and LILRB2). Altogether, these data demonstrate the ability of IOS-1002 to bind and potentially block the LILRB1 and LILRB2 receptors as a checkpoint blockade inhibitor (CPI) of the innate immune system.

IOS-1002 demonstrated binding to human LILRB1, LILRB2 and KIR3DL1 by the HLA domain and interaction of FcγRs through the IgG4 Fc region. IOS-1002 binding affinity to its three innate receptors mimics the receptor binding affinity of natural HLA-receptor interactions and is in clear contrast to the high affinity of monoclonal antibodies targeting LILRB1 or LILRB2. However, when all target receptors are expressed at the cellular level, a significant increase in IOS-1002 binding affinities was observed, which could not be explained by the sum of the single moiety binding affinities, hinting towards an avidity effect. Since the FcγR CD64 showed the highest single receptor binding affinity for IOS-1002, we speculated that IOS-1002 binds to this receptor on primary immune cells and activates associated cells, e.g., macrophages, while binding to LILRB target receptors block the suppressive effect of ITIM-associated receptors on cellular effector functions. This effect was indeed observed once Fc receptor binding was abrogated for IOS-1002, indicating the requirement of an active Fc to enhance avidity to receptors and activity of the drug. In addition, we compared the overall modulation of SHP1/SHP2 phosphorylation in macrophages demonstrating that IOS-1002 alone can indeed downmodulate ITIM signaling in the absence of a secondary stimulus. In macrophages and monocytes, ITIM-mediated signaling pathways of LILRB1 and LILRB2 lead to the suppression of activating signals though the recruitment, phosphorylation and activation of SHP-1 and SHP-2 phosphatases. Activation of SHP-1/SHP-2 can lead to a reduced pro-inflammatory cytokine production, polarization to tolerogenic phenotypes (e.g., M2), reduced phagocytosis and modulation of antigen presentation [12,37,38]. By comparing IOS-1002 efficacy and signaling in macrophage phagocytosis with other LILRB1/LILRB2 blocking antibodies we demonstrated that solely antagonizing these receptors is not sufficient to activate phagocytosis nor downmodulate ITIM signaling. As it has been reported for other macrophage checkpoint therapies e.g., CD47-SIRPa, blockade of this axis alone, in most cases, is not sufficient for the induction of phagocytosis and subsequent antitumor activity [39]. A secondary stimulus, such as other prophagocytic signal (i.e., calreticulin, SLAMF7 and macrophage-1 antigen) or an opsonizing antibody (i.e., rituximab) is required for full activation of macrophages [39,40]. This seems to hold true for high-affinity anti LILRB1/LILRB2 antibodies, compared to IOS-1002 showing superior macrophage phagocytosis and downmodulation of SHP1/SHP2 phosphorylation, without opsonization and prior-activation of cells. Several studies recognize activating ITAM signals as a pre-requisite for initiation of ITIM signaling [41]. In this case, upon CD64 engagement, ITAM-associated kinases will provide phosphate groups to tyrosine residues of ITIM cytoplasmic tail which in turn may downregulate ITAM. As consequence, these cells have activated ‘’eat me’’ signal [39] and blocking ITIM signaling will further increase effector function of the cells and trigger phagocytosis. Taken together, naturally tailored affinities of IOS-1002 toward LILRB receptors accompanied by binding to CD64 grants IOS-1002 a unique mechanism and mode of action in macrophage phagocytosis. Based on our data, tumors with the capacity to recruit monocytes that differentiate into tumor associated macrophages (TAMs) might exert an increased anti-tumoral role in the presence of IOS-1002.

The ability of the IOS-1002 molecule to target LILRB1 and LILRB2 receptors simultaneously is an advantage that increases the pattern of immune cell activation and decreases the likelihood of tumor resistance by receptor substitution. Tumor expressing HLA-G family members bind to both LILRB1 and LILRB2 receptors suggesting that monotherapy blockade of either LILRB1 or LILRB2 alone could lead to tumor escape mechanisms. Moreover, IOS-1002 adds another important mechanism since it binds to KIR3DL1 present on NK cells and leads to increased NK cell activation resulting in enhanced tumor cell killing. The synergistic activity of NK cell activation and PD-1/PD-L1 blockade has recently been demonstrated for the combination of the NKG2A targeting antibody Monalizumab with the PD-L1 targeting antibody Durvalumab in patients with advanced NSCLC [42]. The addition of Monalizumab to Durvalumab almost doubled the response rates and significantly prolonged the progression free survival when compared to Durvalumab alone [42].

Currently, multiple LILRB1 and LILRB2 blocking agents are under evaluation at both the pre-clinical and clinical stage [34,35]. Pre-clinical in vivo and ex vivo data for an anti-LILRB1 blocking antibody (BND-22) demonstrated a shift in the tumor microenvironment towards a more pro-inflammatory state, which translated into tumor suppression seen in different in vivo models including lung, colon and H&N cancer [34]. The most advanced LILRB2 antagonistic antibody (MK-4830) has already passed a phase I dose escalation study with no dose-limiting toxicity and demonstrated clinical benefit in a phase II study when used as monotherapy or—even more pronounced—combined with the PD-1 antibody pembrolizumab [35]. IOS-1002 monotherapy demonstrated anti-tumor efficacy in ex vivo samples derived from cancer patients, while anti-PD-1 monotherapy did not show efficacy in this setting. Considering the high number of patients that either do not respond initially to PD-L1/PD-1 treatment or relapse following PD-L1/PD-1 therapy [43], IOS-1002 might present a suitable combination strategy for improvement of PD-1 antibody treatment.

Since our selection process for HLA molecules was based on their linkage to autoimmunity, one major concern could be the induction of increased autoimmune adverse events as seen for all CPI. Our non-clinical assessment of IOS-1002 included a careful analysis of the potential for IOS-1002 to mediate severe immunological side effects such as cytokine storm-like symptoms and off-target identification. However, no significant cytokine production was detected in primary PBMMC in vitro assays and no off-target binding was identified by cell-microarray technology. In vivo, relevant adverse events, nor any anti-drug antibodies were observed in our non-human primate safety studies, either after single or multiple dosing. This safety profile is supported by the phase II clinical data for MK-4830 monotherapy since no relevant treatment related adverse events were observed. In the anti-LILRB2 and pembrolizumab combination, adverse events occurred at a frequency and severity consistent with anti-PD-1 monotherapy [35]. Due to the reported association of HLA-B57 with autoimmune diseases, one of the concerns of developing IOS-1002 as anti-cancer drug could be induction of autoimmunity. In our non-human primate safety studies, no animal experienced any sign of autoimmunity. Beside the fact that the duration of the study was rather short, one could assume that multiple factors such as naturally tailored affinities to the receptors or lack of loaded peptide in the HLA moiety could explain the lack of excessive immune cell activation. Current cancer immunotherapies predominantly target T cells for tumor eradication, but this often leads to T cell exhaustion and diminished drug efficacy over time. IOS-1002, with its primary focus on myeloid cells, offers a promising alternative. Patients with myeloid-abundant cancers might be the most likely to benefit from this innovative therapy, highlighting the potential for improved outcomes through targeted patient stratification.

In summary, IOS-1002 represents a groundbreaking approach in cancer treatment, designed to stimulate multiple effector cells from both the innate and adaptive immune systems. By interacting with the LILRB1, LILRB2, KIR3DL1 and CD64 receptors, it enhances anti-tumor activity. As a first-in-class, multi-target and multi-functional agent, IOS-1002 is currently being evaluated in a first-in-human phase I trial for various cancer indications.

## 5. Conclusions

In this study, we show that IOS-1002, an HLA-B57-Fc fusion protein, is a first-in-class, multi-target, and multi-functional agent with the ability to activate primary immune cells. IOS-1002 showed significant efficacy in reducing tumor burden in both in vitro and ex vivo models, demonstrated increased activity versus competitor therapies and was shown to be safe in animal models. IOS-1002 is currently being evaluated in a first-in-human phase I trial for various cancer indications as a monotherapy and in combination with an anti PD-1 antibody.

## Data Availability

Data are available on reasonable request.

## Supplementary Materials

The following supporting information can be downloaded at: https://www.mdpi.com/article/10.3390/cancers16162902/s1, Figure S1: (A) Protein expression titers for HLA-B57.B2m and HLA-B57(A46E/V97R).B2m constructs co-transfected and co-expressed in CHO cells with varying ratios of the cognate B2m molecule. (B) SEC-FPLC profiles of HLA-B57.B2m-Fc and HLA-B57(A46E/V97R).B2m-Fc proteins after initial protein A capture. HMW: high molecular weight; LMW: low molecular weight. (C) Quantification of the binding affinities of IOS-1002 with LILRA1-6 and LILRB3-5 surface receptors determined by BLI.(D) Quantification of the binding affinities of IOS-1002 with LILRB1 (*n* = 3), LILRB2 (*n* = 3) and KIR3DL1 (*n* = 3) surface receptors, determined by ELISA. EC50 values are generated from mean ± standard deviation. (E) Quantification of the binding affinities of IOS-1002 to KIR3DL1 using steady state fit (*n* = 4) determined by SPR. (F) Binding of IOS-1002 to CHO cells expressing LILRB1, LILRB2 or KIR3DL1 receptors (*n* = 2, one representative experiment is presented). A 4P-L curve was interpolated for quantification of the EC50. gMFI: geometric mean of fluorescence intensity. (G) Quantification of the binding affinities of IOS-1002 to FcγRIIa (CD32a) and FcγRIIb (CD32b) determined by SPR (*n* = 1). The specified n indicates the number of independent experiments; Figure S2: (A) Binding of a dual anti-LILRB1/2 antibody on different Fc backbones to CHO cells expressing LILRB1, LILRB2 and CD64 FcγRI receptors (*n* = 2, one representative experiment is presented). (B) The expression of LILRB1, LILRB2, and KIR3DL1 on monocytes and macrophages. The isotype of each antibody was used as a negative control (*n* = 4 independent donors). Mean ± standard deviation is presented. (C) Abrogated binding of 500 nM of IOS-1002 binding to LILRB1 and LILRB2 after pre-blocking with 125 nM each of anti-LILRB1 (left) and anti-LILRB2 (right) antibodies (*n* = 1). (D) Simple Western analysis showing expression and phosphorylation of ITIM-associated phosphatases, SHP-1 and SHP-2 in human primary monocytes-derived. The number below the blots indicates the AUC of phosphorylated proteins divided by the AUC of the total protein and subsequently normalized over IgG4 control; Figure S3: (A) Percentage of viability of MDSCs upon treatment with compounds (all 20 μg/mL; *n* = 3). (B) Representative kinetic (*n* = 1) (IncuCyte) and images from live-cell microscopy phagocytosis assays of pHrodo red+ H460 cells and macrophages treated with 428 nM of IOS-1002 (above) or 428 nM of HLA IgG4 (below) at 12 h. Phagocyted cancer cells are quantified by segmentation of the red signal within the macrophages (pHrodo). BF: bright field. (C) Macrophage phagocytosis in the presence of IOS-1002 and anti LILRB1/toward H460 cell line (*n* = 4). Mean ± standard deviation is shown. Statistical analysis was performed with one-way ANOVA and Dunnett’s multiple comparisons test. (D) Macrophage phagocytosis in different cell lines. MIA Paca-2: pancreatic cancer, *n* = 3; RPMI-8226: multiple myeloma, *n* = 3. Comparison to modulator of macrophage phagocytosis (SIRPa-Fc fusion protein) is shown. All indicated compounds were used at a concentration of 20 μg/mL. Statistical analysis was performed with one-way ANOVA and Dunnett’s multiple comparisons test (RPMI-8226 and MIA PaCa-2). * *p* < 0.05, ** *p* < 0.01, *** *p* < 0.001, **** *p* < 0.0001. ns, non-significant. The specified n indicates the number of independent donors; Figure S4: Proliferation of T cells upon IOS-1002 treatment. Non-activated T cells were incubated with MIA PaCa-2 (left) and H1703 (right) cancer cell lines in a cell-cell contact manner and co-cultures were monitored for 72 h. Number of T cells over time (shown as fold changes over timepoint 0) were measured in IncuCyte live-cell microscopy.
