# Supplementary material for: IOS-1002, a Stabilized HLA-B57 Open Format, Exerts Potent Anti-Tumor Activity
Source: Cancers (Basel). 2024 Aug 21;16(16):2902. doi: 10.3390/cancers16162902 (PMC11352577; doi:10.3390/cancers16162902)
Supplement: Supplementary file 1 [file cancers-16-02902-s001.zip › cancers-3128549-supplementary.pdf]

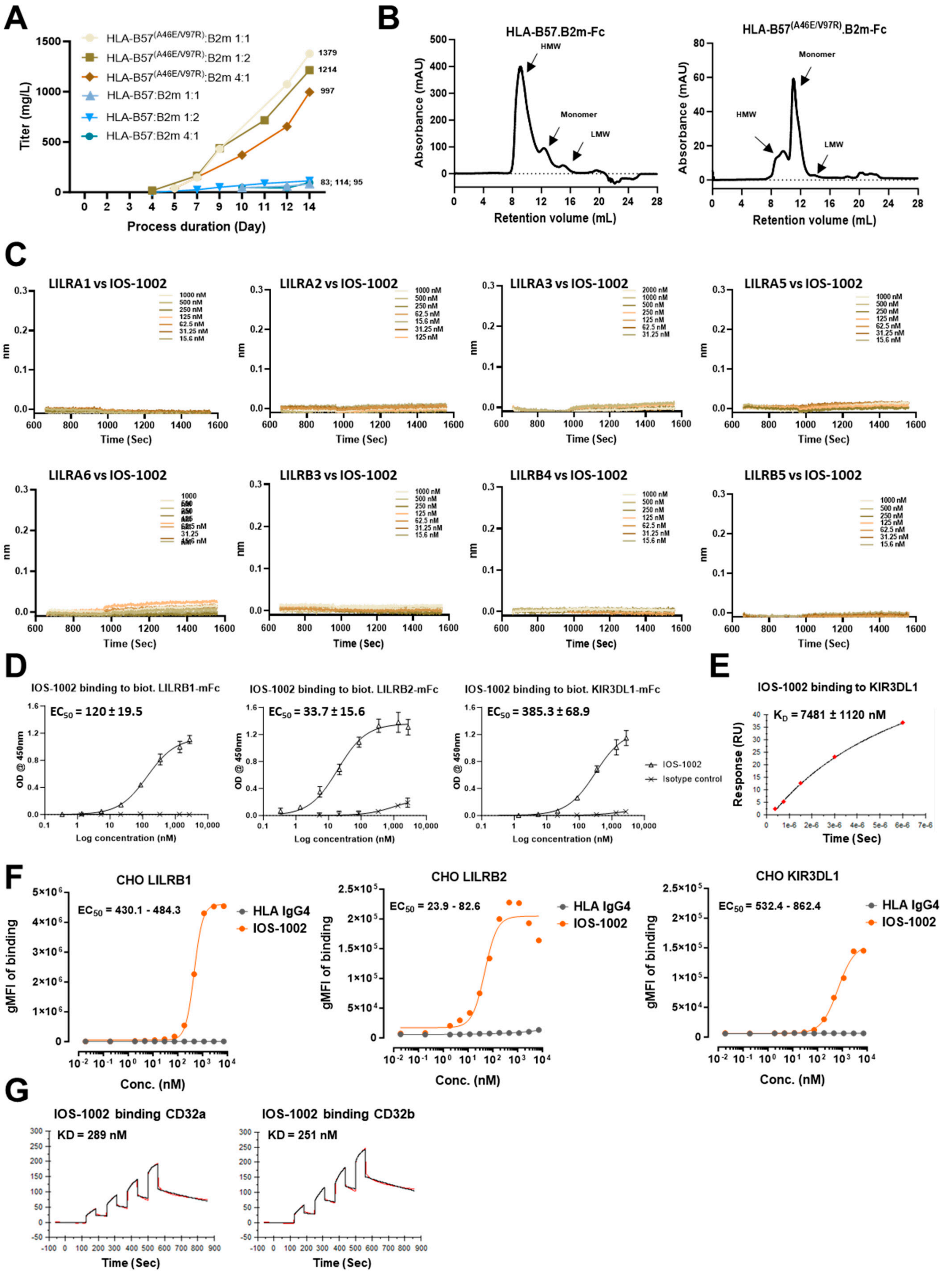
A
HLA-B57(A46E/V97R):B2m 1:1
HLA-B57(A46E/V97R):B2m 1:2
HLA-B57(A46E/V97R):B2m 4:1
HLA-B57:B2m 1:1
HLA-B57:B2m 1:2
HLA-B57:B2m 4:1
1379
1214
997
83; 114; 95
Titer (mg/L)
Process duration (Day)
B
HLA-B57.B2m-Fc
HMW
Monomer
LMW
Absorbance (mAU)
Retention volume (mL)
HLA-B57(A46E/V97R).B2m-Fc
HMW
Monomer
LMW
Absorbance (mAU)
Retention volume (mL)
C
LILRA1 vs IOS-1002
LILRA2 vs IOS-1002
LILRA3 vs IOS-1002
LILRA5 vs IOS-1002
LILRA6 vs IOS-1002
LILRB3 vs IOS-1002
LILRB4 vs IOS-1002
LILRB5 vs IOS-1002
nm
Time (Sec)
D
IOS-1002 binding to biot. LILRB1-mFc
EC50 = 120 ± 19.5
IOS-1002 binding to biot. LILRB2-mFc
EC50 = 33.7 ± 15.6
IOS-1002 binding to biot. KIR3DL1-mFc
EC50 = 385.3 ± 68.9
OD @ 450nm
Log concentration (nM)
IOS-1002
Isotype control
E
IOS-1002 binding to KIR3DL1
KD = 7481 ± 1120 nM
Response (RU)
Time (Sec)
F
CHO LILRB1
EC50 = 430.1 - 484.3
CHO LILRB2
EC50 = 23.9 - 82.6
CHO KIR3DL1
EC50 = 532.4 - 862.4
HLA IgG4
IOS-1002
gMFI of binding
Conc. (nM)
G
IOS-1002 binding CD32a
KD = 289 nM
IOS-1002 binding CD32b
KD = 251 nM
Time (Sec)

Supplementary Figure S1.

(A) Protein expression titers for HLA-B57.B2m and HLA-B57$^{(A46E/V97R)}$.B2m constructs co-transfected and co-expressed in CHO cells with varying ratios of the cognate B2m molecule. **(B)** SEC-FPLC profiles of HLA-B57.B2m-Fc and HLA-B57$^{(A46E/V97R)}$.B2m-Fc proteins after initial protein A capture. HMW: high molecular weight; LMW: low molecular weight. **(C)** Quantification of the binding affinities of IOS-1002 with LILRA1-6 and LILRB3-5 surface receptors determined by BLI. **(D)** Quantification of the binding affinities of IOS-1002 with LILRB1 (n=3), LILRB2 (n=3) and KIR3DL1 (n=3) surface receptors, determined by ELISA. EC_{50} values are generated from mean ± standard deviation. **(E)** Quantification of the binding affinities of IOS-1002 to KIR3DL1 using steady state fit (n=4) determined by SPR. **(F)** Binding of IOS-1002 to CHO cells expressing LILRB1, LILRB2 or KIR3DL1 receptors (n=2, one representative experiment is presented). A 4P-L curve was interpolated for quantification of the EC_{50}. gMFI: geometric mean of fluorescence intensity. **(G)** Quantification of the binding affinities of IOS-1002 to FcγRIIa (CD32a) and FcγRIIb (CD32b) determined by SPR (n=1). The specified n indicates the number of independent experiments.

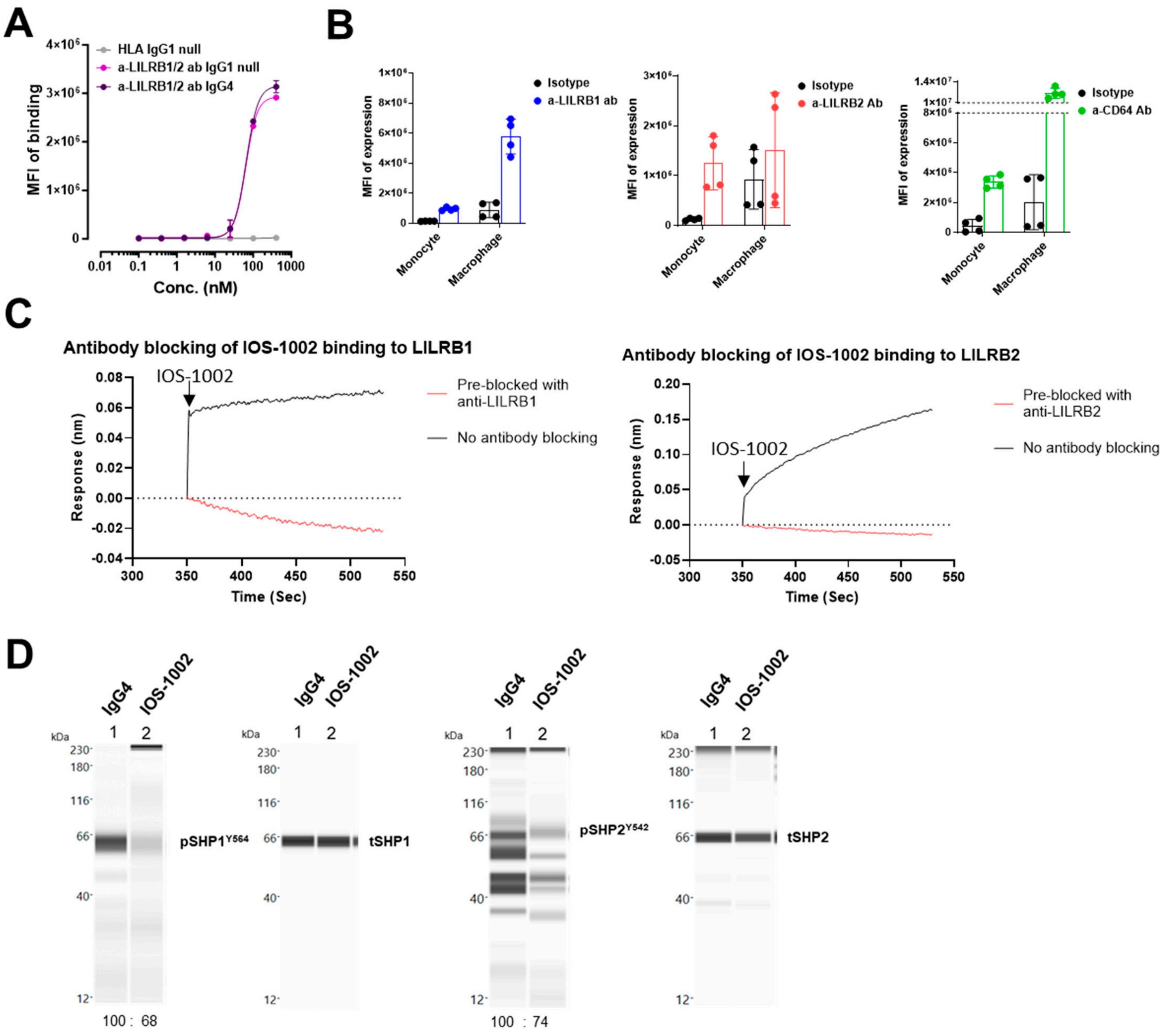

Supplemental Figure S2.

(A) Binding of a dual anti-LILRB1/2 antibody on different Fc backbones to CHO cells expressing LILRB1, LILRB2 and CD64 FcγRI receptors (n=2, one representative experiment is presented). **(B)** The expression of LILRB1, LILRB2, and KIR3DL1 on monocytes and macrophages. The isotype of each antibody was used as a negative control (n=4 independent donors). Mean ± standard deviation is presented. **(C)** Abrogated binding of 500 nM of IOS-1002 binding to LILRB1 and LILRB2 after pre-blocking with 125 nM each of anti-LILRB1 (left) and anti-LILRB2 (right) antibodies (n=1). **(D)** Simple Western analysis showing expression and phosphorylation of ITIM-associated phosphatases, SHP-1 and SHP-2 in human primary monocytes-derived. The number below the blots indicates the AUC of phosphorylated proteins divided by the AUC of the total protein and subsequently normalized over IgG4 control.

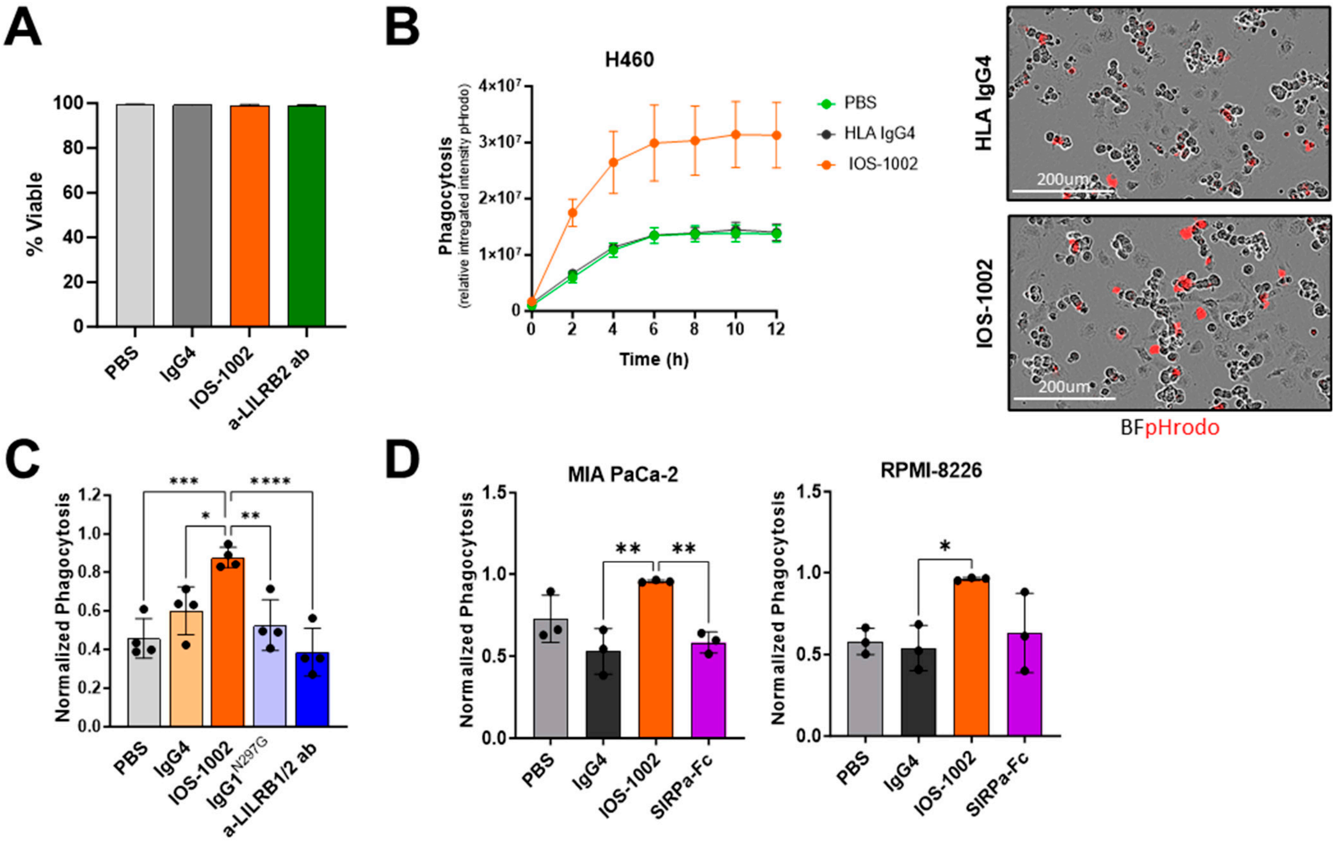

Supplemental Figure S3.

(A) Percentage of viability of MDSCs upon treatment with compounds (all 20ug/ml; n=3). **(B)** Representative kinetic (n=1) (IncuCyte) and images from live-cell microscopy phagocytosis assays of pHrodo red^{+} H460 cells and macrophages treated with 428nM of IOS-1002 (above) or 428nM of HLA IgG4 (below) at 12h. Phagocyted cancer cells are quantified by segmentation of the red signal within the macrophages (pHrodo). BF: bright field. **(C)** Macrophage phagocytosis in the presence of IOS-1002 and anti LILRB1/ toward H460 cell line (n=4). Mean ± standard deviation is shown. Statistical analysis was performed with one-way ANOVA and Dunnett's multiple comparisons test. **(D)** Macrophage phagocytosis in different cell lines. MIA Paca-2: pancreatic cancer, n=3; RPMI-8226: multiple myeloma, n=3. Comparison to modulator of macrophage phagocytosis (SIRPa-Fc fusion protein) is shown. All indicated compounds were used at a concentration of 20ug/ml. Statistical analysis was performed with one-way ANOVA and Dunnett's multiple comparisons test (RPMI-8226 and MIA PaCa-2). *P < 0.05, **P < 0.01, ***P < 0.001, ****P < 0.0001. ns, non-significant. The specified n indicates the number of independent donors.

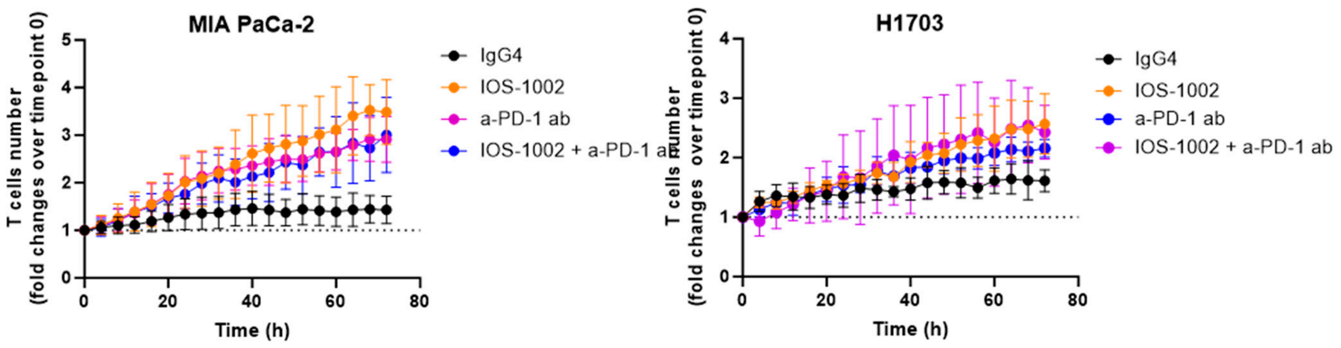

Supplemental Figure S4.

Proliferation of T cells upon IOS-1002 treatment. Non-activated T cells were incubated with MIA PaCa-2 (left) and H1703 (right) cancer cell lines in a cell-cell contact manner and co-cultures were monitored for 72h. Number of T cells over time (shown as fold changes over timepoint 0) were measured in IncuCyte live-cell microscopy.
